# Supplementary material for: A Scoping Review of the Validity, Reliability and Conceptual Alignment of Food Literacy Measures for Adults
Source: Nutrients. 2019 Apr 8;11(4):801. doi: 10.3390/nu11040801 (PMC6520792; doi:10.3390/nu11040801)
Supplement: Supplementary file 1 [file nutrients-11-00801-s001.pdf]

Supplementary material

Table S1. The coding of each question within reviewed food literacy measurement tool against the four domains and eleven components of food literacy as conceptualised by Vidgen and Gallegos (2014).

First author (Year), Country	Tool item and its coding against the eleven components of food literacy as conceptualised by Vidgen and Gallegos (2014)
Papers reporting the development of a tool to explicitly measure food literacy or a part there of	
Begley (2018), Australia[1]	How often have you done the following actions in the last month? Never, Sometimes, Most of the Time, Always<br>1. Plan meals ahead of time (1.2)<br>2. Make a list before you go shopping (1.2)<br>3. Plan meals to include all food groups (1.2)<br>4. Think about healthy choices when deciding what to eat (4.1)<br>5. Feel confident about managing money to buy healthy food (1.3)<br>6. Use Nutrition Information Panel to make food choices (2.2)<br>7. Use other parts of food label to make food choices (2.2)<br>8. Cook meals at home using healthy ingredients (3.1)<br>9. Feel confident about cooking a variety of healthy meals (3.1)<br>10. Try a new recipe (3.1)<br>11. Change recipes to make them healthier (3.1)<br>12. Compare prices of foods to find the best prices on healthy foods (1.3)<br>13. Run out of money for food (1.1)<br>14. Thaw meat at room temperature (3.2)
Krause (2018) Switzerland[2]	Food literacy survey:

First author (Year), Country	Tool item and its coding against the eleven components of food literacy as conceptualised by Vidgen and Gallegos (2014)
	**1.** When I have questions on healthy nutrition, I know where I can find information on this issue. Disagree strongly (1) to Agree strongly (4); I do not have experience with these issues (0)(2.2)<br>**2.** In general, how well do you understand the following types of nutritional information? Very bad (1) to Very good (5); I do not make use of this kind of information (0)<br>▪ Nutrition information leaflets (2.2)<br>▪ Food label information (2.2)<br>▪ TV or radio program on nutrition (2.2)<br>▪ Oral recommendations regarding nutrition from professionals (2.2)<br>▪ Nutrition advice from family members or friends (2.2)<br>**3.** How familiar are you with the Swiss Food Pyramid? Very bad (1) to Very good (5) (4.2)<br>**4.** I know the official Swiss recommendations about fruit and vegetable consumption. Disagree strongly (1) to Agree strongly (4) (4.2)<br>**5.** I know the official Swiss recommendations about salt intake. Disagree strongly (1) to Agree strongly (4) (4.2)<br>**6.** Think about a usual day: how easy or difficult is it for you to compose a balanced meal at home? Very hard (1) to very easy (4); not applicable (0) (4.2)<br>**7.** In the past, how often were you able to help your family members or a friend if they had questions concerning nutritional issues? Never (1) to always (5); there have never been any questions (0) (4.2)<br>**8.** There is a lot of information available on healthy nutrition today. How well do you manage to choose the information relevant to you? Very bad (1) to Very good (5); I have not been interested in these issues (0) (4.2)<br>**9.** How easy is it for you to judge if media information on nutritional issues can be trusted? Very difficult (1) to very easy (4) (4.2)<br>**10.** Commercials often relate foods with health. How easy is it for you to judge if the presented associations are appropriate or not? Very hard (1) to very easy (4) (4.2)

First author (Year), Country	Tool item and its coding against the eleven components of food literacy as conceptualised by Vidgen and Gallegos (2014)
	**11.** How easy is it for you to evaluate if a specific food is relevant for a healthy diet? Very hard (1) to very easy (4) (4.2)<br>**12.** How easy is it for you to evaluate the longer-term impact of your dietary habits on your health? Very hard (1) to very easy (4) (4.2)<br><br>Nutrition knowledge survey:<br>13 What is the healthy plate model? Select one of three images that depict the different proportions of vegetable, protein, and carbohydrate/starchy food in the healthy plate model.) (4.2)<br>14 What is the recommended maximum amount of daily salt consumption? Four options ranging from 5 to 15 g of salt per day. (4.2)
Lahne (2017) USA[3]	Food Self-Efficacy<br>1. I feel limited by my lack of cooking knowledge (3.1)<br>2. I can always manage to decide what I would like to eat at any given time. (1.3)<br>3. When preparing food, I am confident that I can deal with unexpected results. (3.1)<br>4. When preparing food, it is easy for me to accomplish my desired results. (3.1)<br>5. In preparing food, I can solve most problems with enough effort. (3.1)<br>6. I find cooking a very fulfilling activity (3.1)<br>7. For me, cooking is just something to get through as quickly as possible. (3.1)<br>8. Compared to other activities, cooking brings me little enjoyment. (3.1)<br>9. If I try making a new type of food and it does not come out right, I usually do not try to make it again. (3.1)<br><br>Individual Skill - Cooking<br>10. I am comfortable preparing food. (3.1)<br>11. I know how to use the kitchen equipment I have. (3.1)

First author (Year), Country	Tool item and its coding against the eleven components of food literacy as conceptualised by Vidgen and Gallegos (2014)
	12. If everything else is equal, I choose to cook rather than have food prepared by someone else. (3.1)<br><br>Individual Skill - Meal Planning<br>13. I am involved in daily meal preparation. (3.1)<br>14. When I shop for food, I know how I will use the ingredients I am purchasing. (2.1)<br>15. I am confident creating meals from the ingredients I have on hand. (3.1)<br>16. Before I start cooking, I usually have a mental plan of all the steps I will need to complete. (1.2)<br>17. I wish that I had more time to plan meals. (1.1)<br><br>Individual Skill - Shopping<br>18. When presented with two similar products to purchase, I feel confident choosing between them. (2.2)<br>19. I know where to find the ingredients I need to prepare a meal. (2.1)<br><br>Holistic<br>20. I think a lot about what I will cook or eat. (1.1)<br>21. I prefer to spend my time on more important things than food. (1.1)<br><br>Individual skill – clean up<br>22. I feel like cooking is a waste of effort. (3.1)<br><br>Stucture<br>23. I am inspired to cook for other people, like my family or friends. (4.3)<br>24. I feel burdened by having to cook for other people, like my family or friends. (4.3)<br>25. I have a hard time finding enough time to prepare the food I'd like to eat. (1.3)

First author (Year), Country	Tool item and its coding against the eleven components of food literacy as conceptualised by Vidgen and Gallegos (2014)
	26. My family responsibilities prevent me from having time to prepare meals. (1.3)<br>27. My social responsibilities prevent me from having the time to prepare meals. (1.3)<br>28. My job responsibilities prevent me from having the time to prepare meals. (1.3)<br><br>7-point Likert scales, with response options from "Strongly Disagree" (coded as "1") to "Strongly Agree" (coded as "7").<br>Items that were theorized to be negatively correlated with Food Agency were coded in reverse.
Palumbo (2017) , Italy[4]	One a scale from 1 (very difficult) to 4 (very easy), how easy do you believe it is to…<br>Planning and Management<br>1. Find information about the nutritional properties of the foods you plan to buy? (2.2)<br>2. Find information about how to prepare the food that you purchased? (2.2)<br>3. Find information on foods to be eaten during a state of illness or disease? (4.1)<br>4. Find information about how to prepare food to be taken during a state of illness or disease? (4.1)<br>5. Understand the information on the different methods of preservation and use of food? (2.2)<br>6. Understand the nutritional label on food products you want to buy? (2.2)<br>7. Understand which diet is most suitable in case of illness or during a disease? (4.1)<br>8. Understand the food/nutritional recommendations provided by your doctor or dietitian? (4.1)<br>9. Use the food/nutritional recommendations to improve your state of health? (4.2)<br>10. Determine whether the nutritional information you collected is suitable for your specific health needs? (4.1)<br>11. Evaluate the pros and cons of a diet that you intend to follow? (4.1)<br>12. Evaluate the convenience of the products that you intend to buy with respect to similar foodstuffs or alternatives? (2.2)<br>13. Assess the reliability of the information collected on the nutritional properties of food that you intend to buy? (2.2)

First author (Year), Country	Tool item and its coding against the eleven components of food literacy as conceptualised by Vidgen and Gallegos (2014)
	14. Use the information on the food to meet your nutritional needs? (2.2)<br>15. Faithfully follow the recipes for the preparation of particular dishes? (3.1)<br>16. In case of lack of ingredients, make innovations in recipes without compromising the results? (3.1)<br>Selection<br>17. Find information on how and when you can consume high-calorie foods? (4.1)<br>18. Find information on how to handle critical situations such as an uncontrollable hunger or desire to consume alcohol? (4.1)<br>19. Look up information on food safety and hygiene practices? (3.2)<br>20. Search information on how to prevent or treat conditions such as overweight by modifying your eating habits? (4.1)<br>21. Understand the useful recommendations to follow a healthy eating pattern and limit the consumption of saturated fats? (4.1)<br>22. Understand the origin of food and the possible importation of food from areas at risk for health? (2.2)<br>23. Understand why food additives or other substances are added to some foods to improve the commercial characteristics? (2.2)<br>24. Evaluate the accuracy of the nutritional information on food packages? (2.2)<br>25. Evaluate the opportunity to consult information and digital resources to obtain greater and more reliable information on foods? (2.2)<br>26. Assess the cost effectiveness of the foods you intend to purchase and consume? (1.3)<br>27. Assess the food preservation status to verify the quality before consumption? (2.3)<br>28. Assess the reliability of food information reported by social media? (2.2)<br>29. Decide if you consume foods not commonly used, but with high antioxidant properties? (4.1)<br>30. Decide whether to consume foods that help prevent the onset of chronic conditions such as overweight, obesity, diabetes, high blood pressure? (4.1)

First author (Year), Country	Tool item and its coding against the eleven components of food literacy as conceptualised by Vidgen and Gallegos (2014)
	Preparation and eating<br>31. Decide whether to use the information provided by the media on the health and nutritional characteristics of the food? (2.2)<br>32. Find information about the type of social and environmental impacts (sustainability, desertification, fairness ...) related to your eating habits? (2.1)<br>33. Learn about food consumption strategies that favor the individual psycho-physical well-being? (4.1)<br>34. Find information on how to change your eating habits to contribute to local territorial development? (2.2)<br>35. Perceive the influence of external factors (social media, fashion, promotional messages) on your consumption behavior? (1.3)<br>36. Be aware of local initiatives aimed at the promotion of healthy eating at the individual and population level? (4.2)<br>37. Understand and use the advice of family members or friends in regards to nutrition? (1.3)<br>38. Understand the information on allergens listed on food packaging? (2.2)<br>39. Understand the information provided by the media about the social and economic consequences linked to the consumption of particular foods (1.3)<br>40. Understand the information on the beneficial health effects of certain types of foods? (4.1)<br>41. Consider how your food choices impact on environmental sustainability? (2.1)<br>42. Assess the impacts of environmental degradation on the quality of the food you use daily? (2.1)<br>43. Assess what eating habits have a greater impact (in economic and social terms) on the external environment? (2.1)<br>44. Decide to follow a diet that meets your nutritional needs and at the same time takes care of the environment? (2.1)

First author (Year), Country	Tool item and its coding against the eleven components of food literacy as conceptualised by Vidgen and Gallegos (2014)
	45. Change your eating habits that may worsen your health condition? (4.1)<br>46. Change your eating choices that express significant impacts on economic and environmental sustainability of its life context? (2.1)<br>47. Actively participate in associations or interest groups to promote greater knowledge on food quality? (2.3)
Poelman (2018) Netherlands[5]	5 point Likert scale (1= not at all/never to 5= yes/always)<br><br>Food preparation skills<br>1. Are you able to prepare fresh vegetables in different ways? For example cooking, steaming or stir frying, or in different dishes? (3.1)<br>2. Do you find it difficult to prepare a meal with more than five fresh ingredients? (3.1)<br>3. Are you able to alter a recipe yourself? For example if you are missing one of the ingredients? (3.1)<br>4. Are you able to prepare fresh fish in different ways? For example grilling, pan frying or stewing, or in different dishes? (3.1)<br>5. Are you able to prepare a meal using fresh ingredients? So without pre-packed and processed foods? (3.1)<br>6. Are you able to see, smell or feel the quality of fresh foods? For example of meat, fish or fruit? (2.3)<br><br>Resilience and resistance<br>7. Are you able to say 'no' to tasty snacks if you want to? For example birthday treats or finger foods? (4.2)<br>8. Imagine that you are at a place where you see and smell tasty foods. Are you able to resist the temptation of buying them? For example at the train station, the petrol station, or at the bakery? (4.2)

First author (Year), Country	Tool item and its coding against the eleven components of food literacy as conceptualised by Vidgen and Gallegos (2014)
	9. Are you able to eat healthily when you feel stressed? (4.2)<br>10. Do you choose foods that are in line with your mood? For example if you are sad or annoyed? (4.2)<br>11. Are you able to eat healthily if the situation deviates from a regular situation? For example when you have unexpected guests or experience time pressure? (1.2)<br>12. Do you eat the total contents of a bag or container of crisps, candies or cookies in one go? (4.2)<br><br>Healthy snack styles<br>13. Do you take along healthy snacks for yourself when you are on the go? For example fruit, cherry-tomatoes, nuts? (1.2)<br>14. Do you eat vegetables as snacks? (4.2)<br>15. Do you eat fruit as a snack? (4.2)<br>16. Do you have healthy snacks for yourself in stock? For example nuts, carrots, cherry tomatoes, or mini cucumbers? (1.2)<br><br>Social and conscious eating<br>17. Do you find it important to eat at the dinner table if you are eating with others? (4.3)<br>18. Do you find it important to eat dinner at the same time if you are with others? (4.3)<br>19. Do you engage in any other activities while eating? For example reading, working, or watching television? (4.3)<br><br>Examining food labels<br>20. Do you compare the calories, fat, sugar or salt content of different products? (2.2)<br>21. Do you check the nutritional labels of products for calories, fat, sugar or salt content? (2.2)

**First author (Year), Country**	**Tool item and its coding against the eleven components of food literacy as conceptualised by Vidgen and Gallegos (2014)**
	Daily food planning<br>22. If you have something to eat, do you take account of what you will eat later that day? (1.2)<br>23. If you have something to eat, do you reflect on what you have eaten earlier that day? (1.2)<br><br>Healthy budgeting<br>24. Do you purchase healthy foods, even if they are a bit more expensive? For example vegetables, fruit, or whole grain products? (1.3)<br>25. Do you purchase healthy food, even if you have limited money? For example vegetables, fruit, or whole grain products? (1.3)<br><br>Healthy food stockpiling<br>26. Do you have 4 or more packages of crisps, pretzels or savoury snacks in stock? Not coded<br>27. Do you have 4 or more packages of candy, cookies or chocolate in stock? (not coded)<br>28. Do you have 4 or more bottles of sugar sweetened beverages or lemonade with sugar in stock? (not coded)<br>29. Do you have 4 or more cartons of fruit juice in stock? (not coded)
Food literacy strategy indicators	
Boucher (2017) Canada[6]	• Percentage of secondary school students in Ontario who earned at least one credit in a course that included a food skills component (not coded)<br>• Personal ability to cook from basic ingredients (3.1)<br>• Number of persons accessing an Eat Right Ontario dietitian (phone or by email) (not coded)

First author (Year), Country	Tool item and its coding against the eleven components of food literacy as conceptualised by Vidgen and Gallegos (2014)
	• Number of dietitians practising in family health teams and community health centres (not coded)
Tools developed to evaluate a food literacy intervention	
Barbour (2016) Australia[7]	5-point Likert scale: 1 (Not confident at all) to 5 (Extremely confident)<br><br>• How confident do you feel about being able to cook from basic ingredients? (3.1)<br>• How confident do you feel about following a simple recipe? (3.1)<br>• How confident do you feel about tasting foods that you have not eaten before? (3.1)<br>• How confident do you feel about preparing and cooking new foods and recipes? (3.1)
Hutchinson (2016) UK[8]	confidence in cooking a healthy meal (0 to 5; 0 being 'not confident at all' and 5 being 'very confident') (3.1)
Wallace (2016) Australia[9]	Attitude Towards Healthy Eating & Healthy Cooking [2 items]: 5-point Likert scale: 'Not Very Important' (1) to 'Very Important' (5)<br>• Healthy eating (4.1)<br>• Healthy cooking (4.1)<br>Confidence in Healthy Eating & Cooking [3 items]: 4-point Likert scale<br>• Confidence cooking (3.1)<br>• Confidence shopping (2.1)<br>• Confidence planning meals (1.2)<br>Nutrition Knowledge (Dementia-Specific) [6 items]: (4.2)<br>• Which of the herbs and spices listed below are thought to play a role in the prevention of dementia? (turmeric, oregano, garlic, basil, rosemary, sage, parsley, ginger)<br>• Categorise the following foods as energy (E) dense or nutrient (N) dense. (coco-cola, whole grain bread, butter, walnuts, skim milk, fruit roll ups, white bread, apples)<br>• Which of the following foods are good sources of resveratrol? (red grapes, white grapes, beer, blueberries, potatoes, red grape juice, bananas, red wine, peanuts, almonds)

<table>
<tr><th>First author (Year), Country</th><th>Tool item and its coding against the eleven components of food literacy as conceptualised by Vidgen and Gallegos (2014)</th></tr>
<tr><td></td><td>

Which of the following foods are good sources of omega 3 and DHA? (red grapes, salmon, walnuts, tuna, blueberries, almonds, mackerel, tea)
Match the antioxidant with the appropriate food sources. (Vitamin C/Vitamin E/Beta-carotene/Selenium/Zinc/Carrots, rock melon, pumpkin/Brazil nuts, fish, seafood/Lean meat, chicken, oysters/Citrus fruit, parsley, berries/Nuts, eggs, wheat germ)
Circle which of the following foods are good sources of polyphenols. (green tea, peanuts, pomegranate, butter, liver pate ,red grapes, apples, oranges, roast beef, chocolate)

</td></tr>
<tr><td colspan="2">Tools to measure food literacy as a characteristic within broader study</td></tr>
<tr><td>Amuta-Jimenez (2018) USA[10]</td><td>

When available, how often do you use menu information on calories in deciding what to order? Never, Rarely, Sometimes, Often and Always (2.2)
The food label above can be found on the back of a container of a pint of ice cream. We would like to know how easy it is to use this information. Use the food label above to answer questions. (2.2)

If you eat the entire container, how many calories will you eat? (2.2)
If you are allowed to eat 60g of carbohydrates as a snack, how much ice cream could you have? (2.2)
Your doctor advises you to reduce the amount of saturated fat each day, which includes 1 serving of ice cream. If you stop eating ice cream, how many grams of saturated fat would you be consuming each day? (4.2)
If you usually eat 2,500 calories in a day, what percentage of your daily value of calories will you be eating if you eat one serving? (4.2)

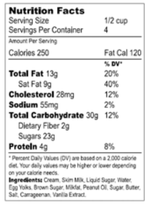

</td></tr>
</table>

First author (Year), Country	Tool item and its coding against the eleven components of food literacy as conceptualised by Vidgen and Gallegos (2014)
Mejean (2017) France[11]	• How often meals are prepared in a typical week (inc preparation of a cold dish or reheating a prepared dish) (two or more times per day, once a day, several times a week but not every day, once a week, less than once a week, never) (3.1)<br>• How much times (in minutes) is usually spent preparing meals, including cooking time (3.1)<br>• Do you enjoy cooking? (yes, including daily meal preparation; yes, but not daily meal preparation, no) (3.1)<br>• Do you wish to cook better (yes/no) (not coded)<br>• Do you wish to cook more often? (Yes/no) (not coded)<br><br>Use of foods with no or minimal processing (from 0 to 12 points) (yes/no) (3.1)<br>Unpeeled, uncut, unprocessed vegetables<br>• Lettuce and other salad, endive, fresh spinach<br>• Beets, carrots, celeriac, etc<br>• Beans, peas, etc<br>• Asparagus, celery, fennel, leeks, artichoke, etc<br>• Broccoli, cauliflower, cabbage, Brussels sprouts, etc<br>• Mushrooms<br>Forms of fish used (4 items)<br>• Use of whole fish not cleaned out (even if participant also used whole fish cleaned out or fish fillets, sliced, pavers or steaks)<br>• Use of whole fish cleaned out (even if participant also used fish fillets, sliced, pavers or steaks)<br>• Breaded fish (even if participant also used whole fish not cleaned out, whole fish cleaned out or fish fillets, sliced, pavers or steaks)<br>• Breaded fish only or none<br>Forms of meat used

First author (Year), Country	Tool item and its coding against the eleven components of food literacy as conceptualised by Vidgen and Gallegos (2014)
	• Chunky uncut pieces, whole poultry not cleaned out, whole poultry cleaned out, cut poultry or meat (even if participant also used ready to cook poultry or meat)<br>• Ready to cook poultry or meat only (ultra-processed met and nuggets) or none<br><br>Cooking skills (from 0 to 41 points) (yes/no) (3.1)<br>Dishes<br>• Make bread<br>• Make bread with a bread maker<br>• Make mashed potatoes (with unprocessed potatoes)<br>• Make instant mashed potatoes<br>• Make savory pie or pizza with homemade pastry shell<br>• Make savory pie or pizza with ready for use pastry shell<br>• Make a vegetable gratin<br>• Make a dish with stewed meat or fish<br>Desserts and pastries<br>• Make ice cream or sorbet with or without ice cream maker<br>• Make yogurt with or without yogurt-maker<br>• Make pancakes or waffles with homemade pancake batter<br>• Make pancakes or waffles with ready for use pancake batter<br>• Make cakes or pastries with homemade pastry shell<br>• Make cakes or pastries with ready for use pastry shell<br>• Make floating islands with homemade custard<br>• Make floating islands with ready for use custard<br>• Make chocolate mousse<br>• Make macaroons<br>• Make pie with homemade pastry shell<br>• Make pie with ready for use pastry shell

First author (Year), Country	Tool item and its coding against the eleven components of food literacy as conceptualised by Vidgen and Gallegos (2014)
	Sauces<br>• Make hollandaise sauce or sauce by reduction (even if participant can make or not salad dressing)<br>• Make 3 or 4 simple sauces* (even if participant can make or not salad dressing)<br>• Make 2 simple sauces (even if participant can make or not salad dressing)<br>• Make only 1 simple sauce (even if participant can make or not salad dressing)<br>• Make salad dressing or none<br>Cooking techniques<br>• Scale and clear out a whole fish<br>• Fillet a whole fish<br>• Stuff meat or poultry<br>• Tie up a roast<br><br>Kitchen equipment 6 items (from 0 to 11 points) (3.1)<br>• Have pressure cooker<br>• Have zester<br>• Have baking pan<br>• Have measuring cup<br>• Have food processor<br>• Have gas oven or electric furnace
Wijayaratne (2018) Australia[12]	Cooking and capability<br>1. I consider myself to be an excellent cook. (3.1)<br>2. Compared to the average person I know a lot about cooking (3.1)<br>3. I have a lot of knowledge about how to cook using different methods (3.1)<br>4. I have a high level of nutrition knowledge (4.2)<br>5. I have a lot of knowledge of how to evaluate the healthiness of food (2.2)<br>6. Compared to the average person, I know a lot about food and nutrition (4.2)

First author (Year), Country	Tool item and its coding against the eleven components of food literacy as conceptualised by Vidgen and Gallegos (2014)
	7. I love preparing food using many different methods (3.1)<br>8. I have all of the kitchen equipment and resources I need to cook meals using different methods (3.1)<br><br>Informed food choices<br>9. Food product information is very important to me. (2.2)<br>10. I always need to know what the product contains before I buy it (2.2)<br>11. I always compare product information labels to decide which food brands to buy (1.3)<br>12. I always compare food product labels to select the healthiest foods (2.2)<br>13. To me the naturalness of the food I buy is an important quality (2.3)<br>14. I always avoid buying food products with additives and preservatives (2.3)<br><br>Making time<br>15. I am not in a hurry these days (not coded)<br>16. Cooking is something that should take time (1.1)<br>17. I always have time to cook (1.1)<br><br>(not) convenience foods and cooking<br>18. I do not use the microwave to cook an evening meal (3.1)<br>19. I do not use many ready to eat foods (3.1)<br>20. I do not choose quick and easy to prepare foods for evening meals (3.1)<br>21. I do not use a lot of ready-made sauces and mixes in the household (3.1)<br><br>Fresh foods focus<br>22. Every meal I cook has at least 3 types of vegetables (4.1)<br>23. Preparing a variety of vegetables at each main meal is an important part of my food preparation (4.1)

First author (Year), Country	Tool item and its coding against the eleven components of food literacy as conceptualised by Vidgen and Gallegos (2014)
	24. I buy mostly fresh foods rather than processed, canned or frozen foods (2.3)<br>25. There are always plenty of fruits and vegetables in my weekly shopping trolley (4.2)<br><br>Planned meals<br>26. I always plan what we are going to eat a couple of days in advance (1.2)<br>27. I always plan meals in advance to ensure they are healthy (1.2)<br>28. I usually decide what food to buy before going to store (1.2)<br>29. Before I go shopping for food, I make a full list of everything needed (1.2)

1. Begley, A.; Paynter, E.; Dhaliwal, S. S., Evaluation Tool Development for Food Literacy Programs. *Nutrients* **2018,** *10* (11).
2. Krause Gréa, C.; Beer-Borst, S.; Sommerhalder, K.; Hayoz, S.; Abel, T., A short food literacy questionnaire (SFLQ) for adults: Findings from a Swiss validation study. *Appetite* **2018,** *120*, 275-280.
3. Lahne, J.; Wolfson, J. A.; Trubek, A., Development of the Cooking and Food Provisioning Action Scale (CAFPAS): A new measurement tool for individual cooking practice. *Food Quality and Preference* **2017,** *62*, 96-105.
4. Palumbo, R.; Annarumma, C.; Adinolfi, P.; Vezzosi, S.; Troiano, E.; Catinello, G.; Manna, R., Crafting and applying a tool to assess food literacy: Findings from a pilot study. *Trends in Food Science & Technology* **2017,** *67*, 173-182.
5. Poelman, M. P.; Dijkstra, S. C.; Sponselee, H.; Kamphuis, C. B. M.; Battjes-Fries, M. C. E.; Gillebaart, M.; Seidell, J. C., Towards the measurement of food literacy with respect to healthy eating: the development and validation of the self perceived food literacy scale among an adult sample in the Netherlands. *The International Journal Of Behavioral Nutrition And Physical Activity* **2018,** *15* (1), 54-54.
6. Boucher, B. A.; Manafò, E.; Boddy, M. R.; Roblin, L.; Truscott, R., The Ontario Food and Nutrition Strategy: identifying indicators of food access and food literacy for early monitoring of the food environment. *Health Promotion And Chronic Disease Prevention In Canada: Research, Policy And Practice* **2017,** *37* (9), 313-319.
7. Barbour, L. R.; Ho, M. Y. L.; Davidson, Z. E.; Palermo, C. E., Challenges and opportunities for measuring the impact of a nutrition programme amongst young people at risk of food insecurity: A pilot study. *Nutrition Bulletin* **2016,** *41* (2), 122-129.
8. Hutchinson, J.; Watt, J. F.; Strachan, E. K.; Cade, J. E., Evaluation of the effectiveness of the Ministry of Food cooking programme on self-reported food consumption and confidence with cooking. *Public Health Nutrition* **2016,** *19* (18), 3417-3427.

9. Wallace, R.; Lo, J.; Devine, A., Tailored nutrition education in the elderly can lead to sustained dietary behaviour change. *Journal of Nutrition, Health and Aging* **2016,** *20* (1), 8-15.
10. Amuta-Jimenez, A. O.; Lo, C.; Talwar, D.; Khan, N.; Barry, A. E., Food Label Literacy and Use among US Adults Diagnosed with Cancer: Results from a National Representative Study. *Journal of Cancer Education* **2018**, 1-10.
11. Mejean, C.; Wendy Si, H.; Gojard, S.; Ducrot, P.; Lampure, A.; Brug, H.; Lien, N.; Nicolaou, M.; Holdsworth, M.; Terragni, L.; Hercberg, S.; Castetbon, K., Social disparities in food preparation behaviours: a DEDIPAC study. *Nutrition Journal* **2017,** *16*.
12. Wijayaratne, S. P.; Reid, M.; Westberg, K.; Worsley, A.; Mavondo, F., Food literacy, healthy eating barriers and household diet. *European Journal of Marketing* **2018**.
